# Neoadjuvant PD-1 blockade with toripalimab with or without celecoxib for patients with mismatch repair-deficient or microsatellite instability-high, locally advanced, colorectal cancer (PICC): long-term outcomes of a single-centre, parallel-group, non-comparative, randomised phase 2 trial

**DOI:** 10.1016/j.eclinm.2025.103499

**Published:** 2025-09-12

**Authors:** Xutao Shen, Yue Cai, Weiwei Li, Lishuo Shi, Jianwei Zhang, Xiaoyu Xie, Zehua Wu, Zhuoxin Zheng, Wuteng Cao, Yanhong Deng, Huabin Hu

**Affiliations:** a Department of Medical Oncology, The Sixth Affiliated Hospital, Sun Yat-sen University, Guangzhou, People's Republic of China; b Guangdong Provincial Key Laboratory of Colorectal and Pelvic Floor Diseases, Guangzhou, People's Republic of China; c Biomedical Innovation Centre, The Sixth Affiliated Hospital, Sun Yat-sen University, Guangzhou, People's Republic of China; d State Key Laboratory of Oncology in South China, Guangzhou, People's Republic of China; e Clinical Research Centre, The Sixth Affiliated Hospital, Sun Yat-sen University, Guangzhou, People's Republic of China; f Department of Radiology, The Sixth Affiliated Hospital, Sun Yat-sen University, Guangzhou, People's Republic of China

**Keywords:** Colorectal cancer, dMMR/MSI-H, Neoadjuvant immunotherapy, Toripalimab, Celecoxib, PD-1 blockade, Long-term survival

## Abstract

**Background:**

Neoadjuvant PD-1 blockade has demonstrated high rates of pathological complete response in patients with mismatch repair-deficient or microsatellite instability-high, locally advanced colorectal cancer. However, prospective data on long-term survival remain limited. Here, we report the 5-year outcomes from the phase 2 PICC study (NCT03926338) evaluating neoadjuvant toripalimab with or without celecoxib in this population.

**Methods:**

The PICC study was a single-centre, open-label, parallel-group, non-comparative, randomised, phase 2 study. A total of 34 patients with stage II–III mismatch repair-deficient or microsatellite instability-high colorectal cancer were enrolled between May 1, 2019, and April 1, 2021, and randomly assigned (1:1) to receive neoadjuvant toripalimab plus celecoxib or toripalimab alone every 14 days for six cycles before surgery. The primary endpoint was pathological complete response, which was previously met. Secondary endpoints included long-term oncologic outcomes, with 5-year overall, cancer-specific, disease-free, and event-free survival assessed in the modified intention-to-treat population. Quality of life was also evaluated as a secondary endpoint in this analysis. Data were analysed with a cutoff date of June 22, 2025.

**Findings:**

Of the 34 patients enrolled, all were assessable and randomised to toripalimab plus celecoxib (n = 17) or toripalimab monotherapy (n = 17). At a median follow-up of 61.9 months (IQR, 55.8–63.6), no disease recurrence was observed in either group. The 5-year overall survival rates were 100% (95% CI 100-100) and 94% (95% CI 84–99) in the combination and monotherapy groups, respectively. Cancer-specific survival at 5 years was 100% (95% CI 100-100) in both groups. The 5-year event-free survival rates were 100% (95% CI 100-100) and 93% (95% CI 80–99), and the 5-year disease-free survival rates were 100% (95% CI 100-100) and 93% (95% CI 80–99), respectively. Adverse events were mostly grade 1–2; two patients experienced grade ≥3 events during the perioperative treatment and two developed asymptomatic hypothyroidism during follow-up. At 3 years post-surgery, both groups reported high QLQ-C30 functional scores (86.7–100.0) and low symptom burden (mostly <10.0). QLQ-CR29 showed similarly preserved anxiety, body image (90.4–98.8), and low-to-moderate sexual interest (16.7–44.4), with low symptom burden (0–16.7).

**Interpretation:**

These results demonstrate encouraging long-term survival outcomes, and suggest ongoing evaluation of a therapeutic neoadjuvant toripalimab strategy, with or without celecoxib, for mismatch repair-deficient or microsatellite instability-high localized colorectal cancer patients.

**Funding:**

This study was supported by the 10.13039/501100001809National Natural Science Foundation of China, the National Key Clinical Discipline of China, the Program of Guangdong Provincial Clinical Research Centre for Digestive Diseases, and the Chinese Society of Clinical Oncology–Junshi Biosciences Oncology Immunity Research Fund.

**Research in context**

**Evidence before this study**

We searched PubMed, without any language restrictions, for studies published up to June 1, 2025, using the terms (“neoadjuvant” OR “preoperative”) AND (“colon cancer” OR “rectal cancer” OR “colorectal cancer”) AND [“mismatch repair-deficient (dMMR)” OR “microsatellite instability-high (MSI-H)”] AND (“immunotherapy” OR “immune checkpoint” OR “PD-1” OR “PD-L1”). We identified several prospective trials and retrospective studies investigating neoadjuvant immunotherapy in patients with mismatch repair-deficient or microsatellite instability-high, locally advanced, colorectal cancer. However, data on long-term survival outcomes in this population remain limited.

**Added value of this study**

To our knowledge, this is the first prospective trial evaluating neoadjuvant single-agent PD-1 blockade in patients with mismatch repair-deficient or microsatellite instability-high, locally advanced, colorectal cancer. Previous analyses demonstrated promising pathological complete response rate with neoadjuvant toripalimab, with or without celecoxib. In this study, we further show that such a strategy may offer sustained long-term benefits. Given the lack of long-term data from other phase 2 trials in this setting, our findings provide timely evidence to inform future clinical practice.

**Implications of all the available evidence**

Our results provide more evidence on the long-term efficacy of neoadjuvant single-agent PD-1 blockade with or without celecoxib in treating mismatch repair-deficient or microsatellite instability-high, locally advanced colorectal cancer. Future prospective multicentre studies with larger cohorts, ideally randomised controlled trials, are needed to validate our findings.

## Introduction

Approximately 15% of non-metastatic colorectal cancers are mismatch repair-deficient or microsatellite instability-high,^1^ which derive minimal benefit from chemotherapy.^2^, ^3^, ^4^ Nevertheless, this molecular subtype shows high sensitivity to immune checkpoint inhibitors in the metastatic setting, likely because these tumours are highly immunogenic and immune infiltrated.^5^ Preclinical studies have further shown that (Cyclooxygenase) COX-2–driven prostaglandin E2 (PGE2) signalling promotes tumour inflammation and suppresses T cell–mediated immunity, and that combining PD-1 blockade with celecoxib enhances antitumour activity in colorectal cancer models.^6^^,^^7^ Based on this rationale, the PICC study was the first prospective trial to evaluate neoadjuvant single-agent PD-1 blockade with or without celecoxib followed by surgery in patients with mismatch repair-deficient or microsatellite instability-high, locally advanced, colorectal cancer. Initial results reported in 2022 showed promising pathological complete response rates of 88% with toripalimab plus celecoxib and 65% with toripalimab monotherapy.^8^

Increasing evidence supports immune checkpoint inhibitors as a promising neoadjuvant strategy and a potential practice-changing therapeutic option for patients with mismatch repair-deficient or microsatellite instability-high, locally advanced, colorectal cancer.^9^ Data from the NICHE-2 and NICHE-3 studies, in which patients with mismatch repair-deficient or microsatellite instability-high, locally advanced, colon cancer were treated preoperatively with dual immune checkpoint inhibitors, nivolumab plus ipilimumab or nivolumab plus relatlimab, showed a similarly high pathological complete response rate of 68%.^10^^,^^11^ In a phase 2 study by Cercek and colleagues, patients with mismatch repair-deficient or microsatellite instability-high stage II or III rectal cancer were given neoadjuvant dostarlimab for 6 months, with chemoradiotherapy and surgery reserved for those with residual disease. Notably, all 49 patients achieved clinical complete response, and the 2-year recurrence-free survival rate reached 96% at a median follow-up of 30.2 months.^12^

However, survival data from prospective clinical trials remain limited, and whether neoadjuvant immunotherapy translates impressive pathological responses into survival advantages has not been fully established. Here, we report the 5-year outcomes from the PICC study, providing the longest follow-up reported to date for this approach.

## Methods

### Study design and participants

The PICC study was an investigator-initiated, single-centre, parallel-group, non-comparative, randomised phase 2 study. Detailed eligibility criteria, study design, and methodology have been published previously.^8^ Briefly, eligible participants were aged 18–75 years, with histologically confirmed colorectal cancer, locally assessed as mismatch repair-deficient or microsatellite instability-high, and clinical stage T3–T4 or any T with lymph node positivity (N+). Key exclusion criteria included metastatic disease (stage IV), non-resectable or recurrent colorectal cancer.

The study was conducted in accordance with the Declaration of Helsinki and the Good Clinical Practice Guideline. The protocol and all amendments were reviewed and approved by the ethics committee of the Sixth Affiliated Hospital, Sun Yat-sen University (approval number: 2019ZSLYEC-149). All patients provided written informed consent. This study is registered with ClinicalTrials.gov, NCT03926338, and remains ongoing.

### Randomisation and masking

Eligible patients were randomly assigned (1:1) to either the toripalimab plus celecoxib group or the toripalimab monotherapy group using a computer-generated sequence prepared by the Clinical Research Centre of the Sixth Affiliated Hospital, Sun Yat-sen University. Patients and treating physicians were not masked to study treatment. Investigators assessing radiologic, endoscopic, and pathologic responses were masked to treatment allocation and all other data.

### Procedures

In both treatment groups, toripalimab (3 mg/kg) was administered intravenously over 30 min on day 1 of each 14-day cycle for a total of six cycles before surgical resection. Patients in the toripalimab plus celecoxib group also received celecoxib (200 mg) orally twice daily from day 1 to day 14 of each cycle. Surgery was scheduled within 4 weeks after the last neoadjuvant toripalimab dose. Postoperative management for both groups was at the investigator’s discretion.

Radiological tumour assessments with chest, abdominal, and pelvic CT scans and/or pelvic MRI were performed at baseline, after the third dose of toripalimab, and preoperatively. Postoperative surveillance included physical exams and serum carcinoembryonic antigen (CEA) every 3 months for years 1–3 and every 6 months for years 4–5. CT scans were done every 6 months for years 1–3 and annually for years 4–5. Colonoscopy was required within the first year, unless preoperatively done, and 3 years postoperatively.

Health-related quality of life (HRQOL) was assessed once 3 years after surgery. No baseline or longitudinal HRQOL data were collected. The assessment was conducted using the validated Chinese versions of the European Organisation for Research and Treatment of Cancer (EORTC) Quality of Life Questionnaire-Core 30 (QLQ-C30, version 3.0) and the colorectal-specific module QLQ-CR29. The QLQ-C30 is composed of 30 ordinal items assessing global health status/QOL (GHS/QOL), 5 functional domains and 9 symptoms. The QLQ-CR29 includes 29 items assessing 4 functional domains and 18 symptoms. HRQOL scores were calculated according to the EORTC recommendations. Higher scores on functional and global health scales indicate better functioning, while higher symptom scores indicate greater symptom burden.

### Outcomes

The primary endpoint was the pathological complete response rate (the proportion of patients with no viable tumour cells in the resected primary tumour sample and all sampled regional lymph nodes) in each treatment group. Key secondary endpoints included overall survival (time from randomisation until death from any cause), cancer-specific survival (time from randomisation to death due to colorectal cancer or to a protocol treatment-related toxicity), event-free survival (time from randomisation until the first occurrence of locally progressive disease leading to an unresectable tumour, local R2 resection, local recurrence after an R0/1 resection, distant metastases, a new primary colorectal cancer, or death from any cause), and disease-free survival (time from surgery until the first occurrence of locoregional recurrence, distant metastases, a new primary colorectal cancer, or death from any cause). HRQOL was also assessed as a secondary endpoint at 3 years post-surgery using the EORTC QLQ-C30 and QLQ-CR29 questionnaires. The pathological outcomes and safety have been reported previously.^8^ This report updates the 5-year overall survival, cancer-specific survival, disease-free survival, event-free survival, and HRQOL.

### Statistical analysis

The primary endpoint was pathological complete response rate. The study was designed to detect a pathological complete response rate of at least 50% in the toripalimab plus celecoxib and/or toripalimab monotherapy group, superior to the historical control rate of 18%, with an estimated power of 82% at a two-sided α significance level of 5%. Efficacy analyses were performed in the modified intention-to-treat population (all patients randomly assigned and treated with at least one dose of toripalimab), with comparisons between treatment groups considered exploratory.

Time-to-event endpoints were analysed using the Kaplan–Meier method to estimate survival probabilities, with corresponding 95% confidence intervals calculated using Greenwood’ s formula. Patients still alive at the time of analysis were censored for overall survival and cancer-specific survival at the last known date alive. Patients without disease events were censored for event-free survival and disease-free survival at the date of their last valid radiological tumour assessment. The median follow-up time was estimated by the reverse Kaplan–Meier method.

HRQOL analysis was performed in the modified intention-to-treat population with available data at 3 years post-surgery. Scores were summarized using means and standard deviations, and between-group comparisons were conducted using the independent t-test.

All statistical analyses were based on available data and performed using IBM SPSS Statistics (version 22.0) and R (version 3.4.1).

### Role of the funding source

The funder provided the study drug toripalimab and had no role in study design, data collection, data analysis, data interpretation, or writing of the report.

## Results

### Patient characteristics

**Fig. 1:**
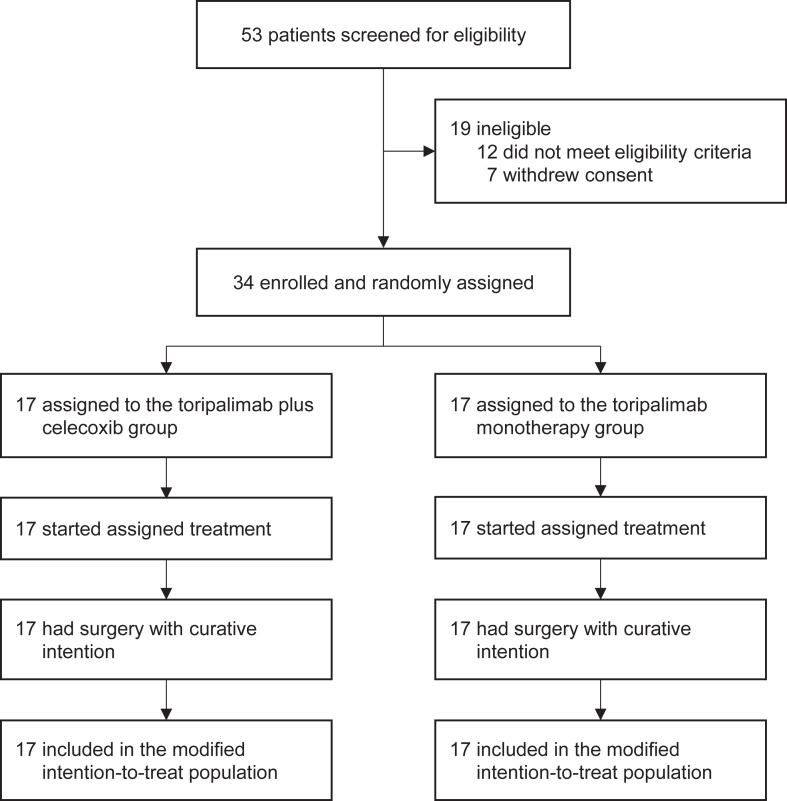
Study profile.

Between May 1, 2019, and April 1, 2021, 34 patients were enrolled and randomly assigned to receive toripalimab plus celecoxib (n = 17) or toripalimab monotherapy (n = 17; Fig. 1). The median age was 49 years (IQR 41–58). Among the 34 patients, 28 (82%) had colon cancer, four (12%) had rectal cancer, and two (6%) had double primary cancers of the colon and rectum. Baseline characteristics were largely comparable between the treatment groups, with a higher proportion of patients in the combination group being female and having suspected Lynch syndrome, clinical T3 stage, and clinical N0 stage (Table 1).

**Table 1:** Baseline characteristics and pathological outcomes.

	Toripalimab plus celecoxib group (n = 17)^a^	Toripalimab alone group (n = 17)
Median age (range), years	45 (35–58)	53 (45–60)
Female sex	8 (47%)	3 (18%)
Suspected Lynch syndrome^b^	4 (24%)	1 (6%)
Primary tumour location		
Right-sided colon	12/19 (63%)	11 (65%)
Left-sided colon	3/19 (16%)	4 (24%)
Rectum	4/19 (21%)	2 (12%)
Clinical T stage		
T3	5/19 (26%)	1 (6%)
T4	14/19 (74%)	16 (94%)
Clinical N stage		
N0	3/19 (16%)	1 (6%)
N1–N2	16/19 (84%)	16 (94%)
Pathological complete response (0% Residual viable tumour)	15 (88%; 95% CI 64–99)	11 (65%; 95% CI 38–86)
Major pathological response (≤10% Residual viable tumour)	16 (94%; 95% CI 71–100)	17 (100%; 95% CI 81–100)
Pathological disease stage		
ypT0N0M0	17/19 (89%)	11 (65%)
ypTisN0M0–0	0	2 (12%)
ypT1N0M0-I	0	1 (6%)
ypT2N0M0-I	0	1 (6%)
ypT3N0M0-IIA	1/19 (5%)	2 (12%)
ypT0N1aM0-IIIA	1/19 (5%)	0

Percentages might not total 100 because of rounding.

a Two patients with both a rectum and an ascending colon tumour are accounted for once in the patient characteristics, and separately per tumour for the tumour-specific features, making a total of 17 patients and 19 tumours in the toripalimab plus celecoxib group.

b Patients were considered to have suspected Lynch syndrome if they met the Amsterdam II criteria.

All patients started assigned treatment and were included in the modified intention-to-treat population (Fig. 1). All underwent surgery and achieved R0 resection. The study met its primary endpoint, with a pathological complete response rate of 88% (15/17) in the toripalimab plus celecoxib group and 65% (11/17) in the toripalimab monotherapy group. One patient in toripalimab plus celecoxib group had resection specimens with tumour-positive lymph nodes (Table 1). All 34 patients received adjuvant toripalimab with or without celecoxib for 3 months at the investigator’s discretion.

### Long-term efficacy

**Fig. 2:**
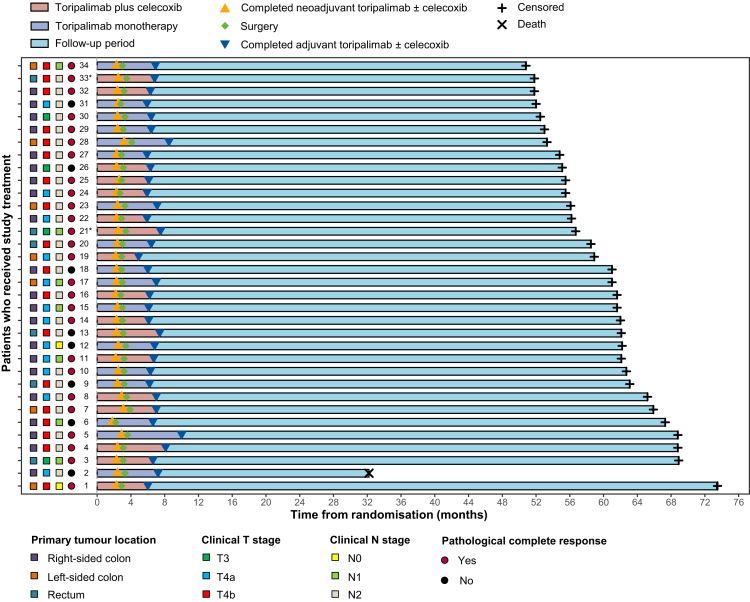
The swimmer plot shows the treatment timeline and long-term follow-up for patients who received study treatment. Each bar represents one patient, annotated with clinical stage, treatment milestones, pathological response, and survival status. ∗Two patients in the toripalimab monotherapy group had synchronous primary mismatch repair-deficient rectum and ascending colon tumours; the highest-stage tumour in rectum was used for analysis in each case.

The data cutoff for this analysis was June 22, 2025. The median follow-up was 61.9 months (IQR, 55.8–63.6). No disease progression occurred during perioperative treatment, and no local or distant recurrences were observed during subsequent follow-up (Fig. 2). One non–cancer-related death occurred in the monotherapy group due to COVID-19 pneumonia (Fig. 2, patient 2). This event was treated as an independent censoring event in the Kaplan–Meier analysis. Given the absence of cancer-related deaths, competing risks methods were not applied for event-free survival or disease-free survival estimation.

**Fig. 3:**
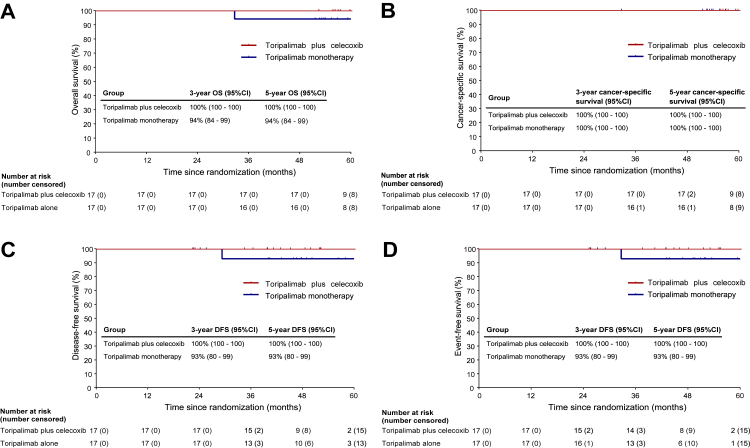
Kaplan–Meier survival estimates in the modified intention-to-treat population. (A) Overall survival, (B) Cancer-specific survival, (C) Event-free survival and (D) Disease-free survival. ∗The survival curves for the two groups completely overlap in Fig. 3B and partially overlap in other Kaplan–Meier plots, reflecting identical or near-identical survival outcomes during certain time intervals.

At the 5-year landmark analysis, the 5-year overall survival rates were 100% (95% CI 100-100) in the combination group and 94% (95% CI 84–99) in the monotherapy group (Fig. 3-A). The cancer-specific survival at 5 years was 100% (95% CI 100-100) in both groups (Fig. 3-B). The 5-year event-free survival rates were 100% (95% CI 100-100) in the toripalimab plus celecoxib group and 93% (95% CI 80–99) in the toripalimab monotherapy group (Fig. 3-C), and the disease-free survival rates at 5 years were similarly 100% (95% CI 100-100) and 93% (95% CI 80–99), respectively (Fig. 3-D).

### Long-term safety

Safety monitoring was continued throughout both the perioperative treatment period and the follow-up phase. Treatment-related adverse events during the neoadjuvant and adjuvant phases have been previously reported in the primary study. In brief, the most common grade 1-2 adverse events included hyperthyroidism, fatigue, rash, and pruritus, with only two patients (one in each group) discontinuing adjuvant therapy due to grade 2 immune-related toxicities. Grade 3 or higher treatment-related events were rare and occurred in two patients: one in the combination group (grade 3 increase in aspartate aminotransferase during neoadjuvant therapy), and one in the monotherapy group (grade 3 elevation in both aspartate and alanine aminotransferase during adjuvant therapy) (Supplementary Table S1). During long-term follow-up, two patients in the toripalimab plus celecoxib group developed asymptomatic hypothyroidism, which was effectively managed with levothyroxine supplementation, maintaining normal thyroid function. No other late immune-related adverse events were observed.

### Health-related quality of life

**Fig. 4:**
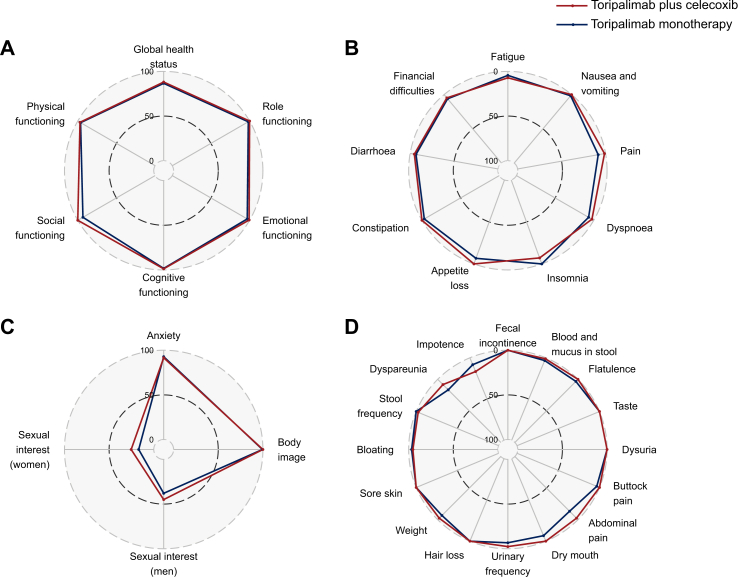
Comparison of EORTC QLQ-C30 and QLQ-CR29 subscales between treatment groups among patients without recurrence or metastasis who completed HRQOL assessments (toripalimab plus celecoxib group, *n* = 14; toripalimab monotherapy group, *n* = 10). (A) QLQ-C30 functional scales. (B) QLQ-C30 symptom scales. (C) QLQ-CR29 functional scales. (D) QLQ-CR29 symptom scales. ∗Scores range from 0 to 100. Higher scores on functional scales reflect better functioning, while higher scores on symptom scales reflect greater symptom burden.

At 3 years post-surgery, HRQOL was assessed using the EORTC QLQ-C30 and QLQ-CR29 questionnaires in 14 patients from the combination group and 10 patients from the monotherapy group. Both groups reported high functional scores on the QLQ-C30, with mean scores ranging from 86.7 to 100.0 on a 100-point scale. Global health status scores were high and comparable, with mean scores of 88.1 in the combination group and 86.7 in the monotherapy group. QLQ-C30 symptom burden was minimal, with most symptom domains showing mean scores <10.0. Similarly, QLQ-CR29 functional domains demonstrated high mean scores in both groups, with mean scores ranging from 90.4 to 98.8 for anxiety and body image, and from 16.7 to 44.4 for sexual interest, while symptom burden was minimal, with most domain-level mean scores between 0 and 16.67. No significant between-group differences were observed across HRQOL functional and symptom domains (Fig. 4).

## Discussion

This updated analysis of the PICC study, with a median follow-up of 61.9 months—the longest reported to date—demonstrates the 5-year survival outcomes of single-agent PD-1 blockade–based neoadjuvant therapy in patients with mismatch repair-deficient or microsatellite instability-high, locally advanced colorectal cancer. These long-term results demonstrate durable efficacy and support the inclusion of toripalimab as a neoadjuvant treatment option in the 2025 NCCN guidelines for mismatch repair-deficient or microsatellite instability-high colorectal cancer.

However, differences in trial design confound determination of the optimal choice between monotherapy and dual immune checkpoint inhibitors, as well as the appropriate duration of neoadjuvant immunotherapy. In the NICHE-2 trial, 4 weeks of nivolumab plus ipilimumab achieved a 68% pathological complete response and 100% 3-year disease-free survival in 111 patients—outcomes comparable to the PICC study using 3 months of single-agent PD-1 blockade–based immunotherapy.^13^ Together, these findings suggest that both short-course doublet immunotherapy and extended monotherapy can yield similar clinical benefit. This differs from the metastatic setting, where dual PD-1/CTLA-4 blockade improves progression free survival compared with single-agent PD-1 blockade.^14^ Although dual immunotherapy may reduce treatment duration, it is associated with higher toxicity,^15^ and global access to anti–CTLA-4 inhibitors remains relatively limited. In contrast, single-agent PD-1 blockade is better tolerated and more widely available, though longer courses may impact treatment compliance. Collectively, balancing efficacy, safety, and treatment compliance remains an important consideration in selecting optimal neoadjuvant strategies.

The unprecedented short-term responses and long-term outcomes observed in these studies have prompted exploration of non-operative management in mismatch repair-deficient or microsatellite instability-high, locally advanced, colorectal cancer. Rectal cancer remains a key context for such strategies, as organ preservation is a long-standing goal due to the potential quality-of-life impairments associated with radical surgery, including permanent stoma formation, sexual dysfunction and low anterior resection syndrome.^16^, ^17^, ^18^ In line with this paradigm, a landmark study by Cercek and colleagues treated 50 patients with mismatch repair–deficient or microsatellite instability–high, locally advanced rectal cancer with 6 months of neoadjuvant dostarlimab; among the 49 who completed therapy, all achieved a clinical complete response and opted for nonoperative management, with a 2-year recurrence-free survival of 96%.^12^ Although results from trials investigating non-operative management using immune checkpoint inhibitors for mismatch repair-deficient or microsatellite instability-high, colon cancer are awaited, the excellent survival observed after surgery in our study and the higher prevalence of this subtype in colon cancer support that non-operative strategies should remain investigational for now. Such an approach may be particularly appropriate for frail or elderly patients, and those who decline surgery after achieving a clinical complete response. Recent findings from the NEOCAP study and a prospective cohort by Cercek and colleagues further support this approach, suggesting that organ preservation may be feasible in colon cancer patients with clinical complete response after neoadjuvant immunotherapy.^12^^,^^19^ However, in patients with Lynch syndrome, for whom prophylactic subtotal or total colectomy remains debated, the watch-and-wait strategy should be approached with caution due to the risk of metachronous colorectal cancers and the lack of evidence that prior PD-1 blockade prevents second primary tumours; intensive surveillance with frequent colonoscopy remains essential.^20^ Further refinement is needed to identify appropriate candidates, potentially aided by circulating tumour DNA (ctDNA), emerging multi-omics profiling, and multimodal assessment including PET-CT, endoscopy, and histopathologic evaluation, which may offer earlier and more precise indicators of treatment response.^12^^,^^21^^,^^22^

In our study, all patients received six months of perioperative immunotherapy. Notably, favourable long-term survival was observed in patients with pathological complete response, as well as in patients without it. Likewise, in NICHE-2, 14 patients (13%) had residual nodal disease, and all remained disease-free, although only three received adjuvant chemotherapy.^23^ These findings highlight ongoing uncertainty regarding the relative contributions of the neoadjuvant and adjuvant immunotherapy components.^24^ Emerging evidence suggests that extending neoadjuvant immunotherapy may increase the incidence of complete response.^25^ This approach of prolonging preoperative therapy has also demonstrated clinical benefit in total neoadjuvant treatment, with improved pathological complete response rates and enhanced long-term survival in patients with locally advanced rectal cancer.^26^ Thus, for patients unlikely to achieve a complete response or those considering non-operative management, extended total neoadjuvant immunotherapy, or combination treatment may offer a more promising strategy. Future trials may focus on comparing and tailoring these different approaches.

Long-term HRQOL outcomes following neoadjuvant immunotherapy in colorectal cancer were favourable. At 3 years post-surgery, patient-reported outcomes indicated sustained quality of life among those treated with neoadjuvant PD-1 blockade. QLQ-C30 functional scores remained high with minimal symptom burden, while QLQ-CR29 showed preserved bowel function and body image. Overall, the patient-reported outcomes was comparable to that of the general population.^27^ In contrast, the FOWARC study have documented persistent defecation-related symptoms following chemotherapy.^28^ These findings support the potential of neoadjuvant toripalimab with or without celecoxib to deliver sustained clinical benefit alongside improved long-term quality of life.

Although the results of our study are promising, the sample size was relatively small, and only a subset of patients completed the 3-year HRQOL assessments, these findings—particularly those related to patient-reported outcomes—should be interpreted with caution and regarded as exploratory, pending confirmation in larger prospective cohorts. In addition, this was a single-centre study, which may limit the generalizability of safety and efficacy findings to broader clinical settings. The larger, multicentre, randomised PICC-2 trial, which investigates 6 months of neoadjuvant toripalimab with or without celecoxib in patients with mismatch repair-deficient or microsatellite instability-high, locally advanced, colorectal cancer, is expected to provide more robust evidence. Furthermore, radiological assessment of lymph node status in mismatch repair-deficient or microsatellite instability-high colon cancer is often inaccurate, frequently leading to clinical overstating and potential overtreatment. Previous studies have reported that up to 27% of patients are classified as cN + but are ultimately pN0, highlighting the limitations of imaging-based staging in this setting.^29^^,^^30^ Additionally, this study did not include a control group undergoing upfront surgery, limiting direct comparisons between neoadjuvant and standard treatment approaches. Notably, prior studies have shown approximately one third of patients with T4 and/or N2 high-risk stage III mismatch repair-deficient or microsatellite instability-high colon cancer experienced disease recurrence or death within 2 years after curative surgery and adjuvant chemotherapy.^31^ In the ATOMIC trial, recurrence occurred in 23% of patients receiving mFOLFOX6 alone, and 13% with mFOLFOX6 plus atezolizumab within 3 years.^24^ These findings highlight the substantial recurrence risk even after adjuvant chemotherapy and immunotherapy. While differences in staging methods—clinical CT-based staging in the neoadjuvant setting versus pathological staging after upfront surgery—may affect comparability, the recurrence-free outcomes observed in our cohort over long-term follow-up remain encouraging and support further investigation of this approach.

In conclusion, the PICC study is the first to report 5-year survival outcomes of neoadjuvant single-agent PD-1 blockade followed by surgery in mismatch repair-deficient or microsatellite instability-high, locally advanced, colorectal cancer, demonstrating encouraging survival and supporting further exploration of neoadjuvant toripalimab with or without celecoxib, as a treatment strategy.

## Contributors

Huabin Hu and Yanhong Deng designed the study and wrote the study protocol. Yue Cai, Weiwei Li, Jianwei Zhang, Xiaoyu Xie, Zehua Wu, Zhuoxin Zheng, Yanhong Deng, and Huabin Hu recruited patients and collected data. Wuteng Cao performed radiological assessment and efficacy evaluation. Xutao Shen, Yanhong Deng, and Huabin Hu accessed and verified the underlying data. Lishuo Shi did the statistical analysis. Xutao Shen and Huabin Hu drafted the initial manuscript. All authors reviewed or revised the manuscript and approved the final version. The corresponding author had full access to all data and had final responsibility for the decision to submit for publication.

## Data sharing statement

De-identified individual participant data (including a data dictionary) that underlie the results reported in this Article will be shared beginning 9 months and ending 36 months following publication. Investigators who wish to use the data for individual patient data meta-analyses should submit their proposal to the corresponding authors. Data access requests will be reviewed by The Sixth Affiliated Hospital, Sun Yat-sen University to ensure there are no intellectual property or confidentiality constraints. Upon approval, data will be made available via an appropriate data archive.

## Declaration of interests

We declare no competing interests.
