# Supplementary material for: Neoadjuvant PD-1 blockade with toripalimab with or without celecoxib for patients with mismatch repair-deficient or microsatellite instability-high, locally advanced, colorectal cancer (PICC): long-term outcomes of a single-centre, parallel-group, non-comparative, randomised phase 2 trial
Source: eClinicalMedicine. 2025 Sep 12;88:103499. doi: 10.1016/j.eclinm.2025.103499 (PMC12572784; doi:10.1016/j.eclinm.2025.103499)
Supplement: Supplementary Table S1 [file mmc1.docx]

**Table S1. Treatment-related adverse events (n=34)**

|  | **Toripalimab plus celecoxib group (n=17)** | | |  | **Toripalimab monotherapy group (n=17)** | | |
| --- | --- | --- | --- | --- | --- | --- | --- |
|  | **Grade 1** | **Grade 2** | **Grade 3** |  | **Grade 1** | **Grade 2** | **Grade 3** |
| **Treatment-related adverse events during the neoadjuvant phase** | | | | | | | |
| Hyperthyroidism | 3 (18%) | 0 | 0 |  | 1 (6%) | 0 | 0 |
| Fatigue | 2 (12%) | 0 | 0 |  | 3 (18%) | 1 (6%) | 0 |
| Aspartate aminotransferase increased | 2 (12%) | 0 | 1 (6%) |  | 2 (12%) | 0 | 0 |
| Abdominal pain | 2 (12%) | 0 | 0 |  | 1 (6%) | 0 | 0 |
| Pruritus | 1 (6%) | 1 (6%) | 0 |  | 2 (12%) | 1 (6%) | 0 |
| Nausea | 1 (6%) | 0 | 0 |  | 3 (18%) | 0 | 0 |
| Rash | 1 (6%) | 0 | 0 |  | 2 (12%) | 1 (6%) | 0 |
| Dizziness | 1 (6%) | 0 | 0 |  | 0 | 0 | 0 |
| Alanine aminotransferase increased | 0 | 1 (6%) | 0 |  | 0 | 1 (6%) | 0 |
| Hypothyroidism | 0 | 1 (6%) | 0 |  | 0 | 0 | 0 |
| Decreased appetite | 0 | 0 | 0 |  | 1 (6%) | 0 | 0 |
| Dry mouth | 0 | 0 | 0 |  | 1 (6%) | 0 | 0 |
| Fever | 0 | 0 | 0 |  | 1 (6%) | 0 | 0 |
| Gamma-glutamyltransferase increased | 0 | 0 | 0 |  | 1 (6%) | 0 | 0 |
| Infusion related reaction | 0 | 0 | 0 |  | 0 | 1 (6%) | 0 |
| **Treatment-related adverse events during the adjuvant phase** | | | | | | | |
| Hyperthyroidism | 3 (18%) | 0 | 0 |  | 0 | 0 | 0 |
| Alanine aminotransferase increased | 2 (12%) | 0 | 0 |  | 1 (6%) | 0 | 1 (6%) |
| Pruritus | 1 (6%) | 0 | 0 |  | 2 (12%) | 1 (6%) | 0 |
| Hypothyroidism | 1 (6%) | 0 | 0 |  | 1 (6%) | 0 | 0 |
| Fatigue | 1 (6%) | 0 | 0 |  | 1 (6%) | 0 | 0 |
| Aspartate aminotransferase increased | 1 (6%) | 0 | 0 |  | 0 | 0 | 1 (6%) |
| Gamma-glutamyltransferase increased | 1 (6%) | 0 | 0 |  | 0 | 0 | 0 |
| Arthralgia | 0 | 1 (6%) | 0 |  | 0 | 0 | 0 |
| Hypophysitis | 0 | 1 (6%) | 0 |  | 0 | 0 | 0 |
| Myalgia | 0 | 1 (6%) | 0 |  | 0 | 0 | 0 |
| Rash | 0 | 0 | 0 |  | 2 (12%) | 1 (6%) | 0 |
| Pneumonitis | 0 | 0 | 0 |  | 0 | 1 (6%) | 0 |

NOTE. Data are n (%) unless otherwise indicated. Some patients had more than one adverse event.

Data cutoff was August 10, 2021. All grade 1-3 treatment-related adverse events are shown. No grade 4-5 treatment-related adverse events were reported.
